# A Review of the Impact of Streptococcal Infections and Antimicrobial Resistance on Human Health

**DOI:** 10.3390/antibiotics13040360

**Published:** 2024-04-15

**Authors:** Raina Gergova, Vasil Boyanov, Adile Muhtarova, Alexandra Alexandrova

**Affiliations:** Department of Medical Microbiology, Medical Faculty, Medical University of Sofia, Zdrave Str. 2, 1431 Sofia, Bulgaria; v.boyanov@medfac.mu-sofia.bg (V.B.); amuhtarova@medfac.mu-sofia.bg (A.M.); alexandrova_sa@medfac.mu-sofia.bg (A.A.)

**Keywords:** *Streptococcus* genus, *Streptococcus pneumoniae*, *Streptococcus pyogenes*, *Streptococcus agalactiae*, antibiotic resistance, mechanisms of resistance

## Abstract

*Streptococcus pneumoniae*, *Streptococcus pyogenes* (GAS), and *Streptococcus agalactiae* (GBS) are bacteria that can cause a range of infections, some of them life-threatening. This review examines the spread of antibiotic resistance and its mechanisms against antibiotics for streptococcal infections. Data on high-level penicillin-resistant invasive pneumococci have been found in Brazil (42.8%) and Japan (77%). The resistance is caused by mutations in genes that encode penicillin-binding proteins. Similarly, GAS and GBS strains reported from Asia, the USA, and Africa have undergone similar transformations in PBPs. Resistance to major alternatives of penicillins, macrolides, and lincosamides has become widespread among pneumococci and streptococci, especially in Asia (70–95%). The combination of several *emm* types with *erm*(B) is associated with the development of high-level macrolide resistance in GAS. Major mechanisms are ribosomal target modifications encoded by *erm* genes, ribosomal alterations, and active efflux pumps that regulate antibiotic entry due to *mef*A/E and *msr*D genes. Tetracycline resistance for streptococci in different countries varied from 22.4% in the USA to 83.7/100% in China, due to *tet* genes. Combined tetracycline/macrolide resistance is usually linked with the insertion of *ermB* into the transposon carrying *tetM*. New quinolone resistance is increasing by between 11.5 and 47.9% in Asia and Europe. The mechanism of quinolone resistance is based on mutations in *gyrA*/*B*, determinants for DNA gyrase, or *parC*/*E* encoding topoisomerase IV. The results for antibiotic resistance are alarming, and urgently call for increased monitoring of this problem and precautionary measures for control to prevent the spread of resistant mutant strains.

## 1. Introduction

The *Streptococcus* genus is a heterogeneous group of Gram-positive bacteria with similar microscopic spherical morphology, mainly classified based on their cell wall surface antigens [1]. This genus comprises various representatives that are a part of the resident microbiota of mucosal membranes of the mouth, upper respiratory and lower genital tract and causative agents of purulent infections that vary in severity from mild throat infections to invasive ones, abscesses, bacteremia, pneumonia, meningitis, and streptococcal toxic shock syndrome. *Streptococcus pneumoniae*, *Streptococcus pyogenes*, and *Streptococcus agalactiae* are responsible for millions of deaths worldwide due to their virulence and damage to human health [2,3,4,5]. The emergence and evolution of the resistance to antimicrobials is a dynamic process and can vary widely among countries over the years and in *S. pneumoniae* strains, streptococci Lancefield groups A (GAS, *S. pyogenes*), B (GBS, *S. agalactiae*), group C (GCS) and G (GGS), as well as oral viridans group streptococci (a rare cause of bacteremia and infective endocarditis). The development of resistance among the streptococcal isolates is a major reason for the failure to eradicate them, and often causes additional complications with the initial infection [6,7,8]. The first line of choice for the treatment of both invasive and noninvasive streptococcal infections remains β-lactam antibiotics, especially penicillins [7]. Most *S. pneumoniae* strains, and all GAS and GBS, are highly sensitive to penicillin, amoxicillin, and some cephalosporins in vitro. However, mutations in gene (*pbp2x*) encoded penicillin-binding proteins (PBPs) corresponding to the beta-lactam resistance, and associated with certain serotypes of pneumococci were detected more often in recent years [3]. The first single GAS and GBS isolates suspected of *pbp2x* gene mutations and lower susceptibility to penicillin were also reported during the last few years [8,9]. Multiple streptococcal isolates quickly become resistant to macrolides and less to lincosamides, which are alternative drugs for patients with an allergy to penicillin, or failed beta-lactam therapy [10]. This resistance toward macrolides in streptococci from various geographical areas has emerged less than 20 years since the discovery and use of the first macrolide erythromycin [6,11,12,13,14,15]. The resistance to tetracyclines is another very common one in many countries, and new problematic resistance to the notable fluoroquinolones has appeared in recent years [3,16,17,18].

The development of antibiotic resistance among the streptococcal isolates is the major cause of treatment failure. According to the World Health Organization (WHO), the emergence and development of antibiotic resistance is one of the greatest threats to human health (https://www.who.int/news-room/fact-sheets/detail/antibiotic-resistance /accessed on 2 April 2024) [19]. That is why it has to be studied, monitored, and analyzed. Various genetic mechanisms of resistance to the most commonly used antimicrobials have been discovered in the last few years [3,9].

The present review aims to comparatively evaluate the epidemiology of streptococcal infections, as well as the recent emergence, development, and geographical spread of antibiotic resistance and encoding genetic elements of the most important antimicrobial agents for the treatment of these problematic infections.

## 2. Overview of Results

**Figure 1:**
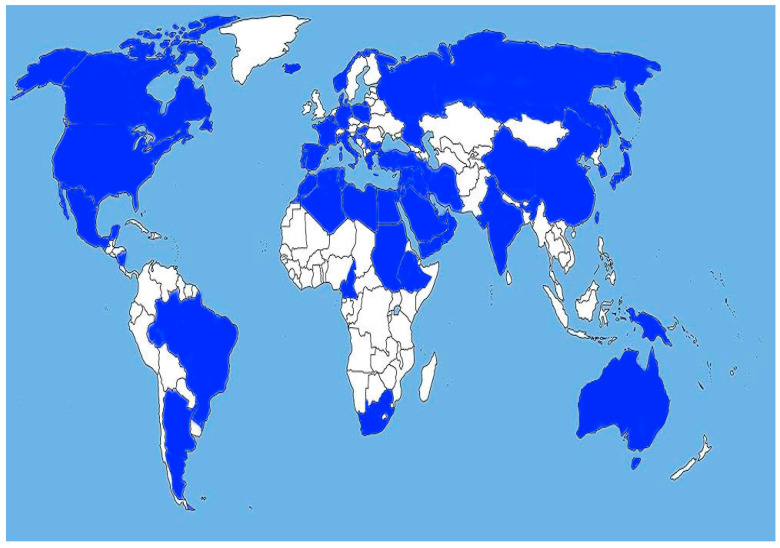
Geographic distribution of included data. The referenced countries are marked blue by the authors.

Extracted information, including geographic location, study period, leading antimicrobial resistance, and reference number, was inserted into Table 1, Table 2 and Table 3 for further analysis. The data from 36 geographic areas including Argentina, Australia, Brazil, Bulgaria, Cameroon, Canada, China, Croatia, Denmark, Ethiopia, France, Germany, Greece, Hungary, Iceland, India, Iran, Italy, Japan, Mexico, Middle East and North Africa (MENA), Nicaragua, New Guinea, Norway, Poland, Portugal, Republic of South Africa, Russia, Serbia, South Korea, Spain, Taiwan, Turkey, and the USA, presented in Figure 1 and Table 1, Table 2 and Table 3, were analyzed and evaluated.

### 2.1. Epidemiology of Streptococcal Infections

#### 2.1.1. Diseases Caused by *S. pneumoniae*

*S. pneumoniae* is the most common bacterial causative agent of a wide range of respiratory tract infections. It has historically been the leading cause of community-acquired pneumonia. The severity of this disease is due to significant virulence of the antiphagocytic effect of capsular polysaccharides and pneumolysin production, as well as a strong inflammatory response triggered by the release of cytokines and activation of complement pathways by cell wall proteins and DNA release following bacterial cell rupture. This pathogen can initially asymptomatically colonize the mucosal membranes of the nasopharynx (up to 50–60% more prevalent in healthy preschool and school-aged children, compared to less than 10–20% of healthy carrier adults) [3,20,21]. Pneumococci can migrate to the lungs where they cause alveolar pneumonia if the pathogen is not cleared by the immune system in certain hosts with increased susceptibility to infection.

In addition to pneumonia, the most common disease worldwide, *S. pneumoniae* causes other severe infections such as bacteremia, sepsis, meningitis, acute purulent otitis media, and sinusitis. In childhood the conjugate vaccination against the most virulent capsular serotypes of *S. pneumoniae* has decreased the colonization frequency, but the WHO estimated that 1.6 million deaths in 2005, including 1 million children under 5 years of age, occurred due to pneumococcal pneumonia, especially since the emergence of multidrug-resistant *S. pneumoniae* [22]. Some authors think that switching between encapsulated and non-encapsulated strains of *S. pneumoniae* may play an essential role in pneumococcal serotype dynamics and the spread of antibiotic resistance. Frequent changes in capsular antigens may partly explain the observed variation in recombination rates and the translation of antibiotic resistance among pneumococcal lineages [23]. The severity of infection increases when pneumococci are combined with Respiratory Syncytial virus in young children [22]. Also, it is a common co-infection in influenza (flu) and COVID-19 patients, and is associated with higher morbidity and mortality [24]. This pathogen causes more than 50% of bacterial meningitis and about 40,000 fatal illnesses annually in the United States. In 2017, the WHO included penicillin-non-susceptible *S. pneumoniae* as one of the 12 most critical pathogens that are a priority for the detection of new antibiotics for the treatment of these types of infections. The resistance of *S. pneumoniae* against ß-lactams especially in combination with macrolide–lincosamide resistance is a serious world problem [19,25].

**Table 1:** Recent data for the distribution of *S. pneumoniae* antibiotic resistance in different countries/regions.

	Country	Target Population	(Study Period) Total n	Antimicrobial Resistance						Ref.
Europe				Pen	Cro	Ery	Cli	Tet	Levo	
	Bulgaria	Non-IPD, children up to 9 years	(2019–2021) n = 147	38.1%	16.3%	58.5%	46.9%	39.5%	-	[16]
	Bulgaria	IPD and non-IPD, all age groups	(2011–2016) n = 198	46.5%	19.7%	43.9%	36.4%	37.4%	1.0%	[26]
	Serbia	Respiratory and IPD isolates from children up to 18 years	(2004–2009) n = 5293	-	-	44.9% (2009)	-	-	-	[27]
	Croatia	IPD, adults	(2005–2019) n = 1108	19.6% IE +R	≤2% IE	23.0%	-	-	0.4%	[28]
	Turkey	Healthy children 0–6 years	(2015) n = 150	14.3%	-	47.7%	52.4%	-	-	[29]
	Poland	Children at 2–5 years with recurrent acute pharyngotonsillitis	(2011) n = 57	45.1%	-	52.9%	51.0%	43.1%	-	[21]
	Spain	IPD, all age groups	(1979–2008) n = 19 266	22.3% (in 2008)	5.9% (in 2005)	26.6% (in children) 20.7% (in adults)	-	-	-	[30]
USA and Latin America	USA	Non-IPD and IPD, all age groups	(2009–2017) n = 7254	13.9–3.8% in period (2009–2017)	11.6–2.8%	37.5–45.2%	19.3–17.0%	22.4–20.8%	-	[31]
	Argentina	Children ≤ 6 years with IPD	2019 (n = 115)	39.7%	2.6%	-	-	-	-	[32]
	Brazil	Retrospective study for patients with IPD	(2007–2012) n = 328	42.8%	18.6%	-	7.9%	-	0.3%	[33]
Asia	Japan	Non-encapsulated SPN	(2011–2019) n = 71	PISP: 33.8%, PRSP: 33.8%	-	94.3%	-	-	-	[34]
	Japan	Pediatric population	(2001, 2004, 2007, 2010, 2012)	64.6%, 67.0%, 56.2%, 76.9% 49.5%,	-	-	-	-	-	[25]
	China	Meta analysis among children with IPD	(2006–2013)	32% (total n = 1345	14.7% (1216)	94.4% (1396)	92.3% (1204)	83.7% (1335)	-	[35]
Africa	Tunisia	Ery-R respiratory and non-respiratory SPN	(2010–2016) n = 86	81.4% PNSP 1.2% high R			64.0%	39.5%	2.32%	[36]
	Ethiopia	Hospital-based prospective study, all age groups	(2018–2019) n = 57	17.5%	1.8%	59.6%	17.5%	38.6%		[37]

Notes: SPN—Streptococcus pneumoniae, IPD—invasive pneumococcal disease, Pen—Penicillin, Cro—Ceftriaxone, Ery—Erythromycin, Cli—Clindamycin, Tet—Tetracycline, Levo–Levofloxacin.

#### 2.1.2. Infections Caused by Beta-Hemolytic Streptococci

##### *S. pyogenes* (GAS) Infections

*S. pyogenes* is a human pathogen that causes more than 700 million infections annually, and 18 million severe GAS diseases worldwide, resulting in about 500,000 deaths occurring each year due to its multiple virulence factors, most of them unique to this species [2,8]. The different combinations of *emm* types and other virulence determinants, encoding various exotoxins and enzymes, evasins and invasins, blocking different steps of immune defense, could indicate fundamental differences in host–pathogen interactions among GAS strains. This could contribute to the variety in the pathogenesis of *S. pyogenes* and clinical manifestations of streptococcal infections [2,38]. The diseases caused by this pathogen are of public health significance and include tonsillo-pharyngitis, scarlet fever, impetigo, erysipelas, cellulitis, bacteremia, streptococcal toxic shock syndrome, and necrotizing fasciitis, as well as complications such as acute rheumatic fever and post-streptococcal glomerulonephritis. Soft tissue GAS invasive infections mostly present with shock and multi-organ failure [5,39]. GAS can infect anyone of any age but is more common and affects preschool children followed by school-aged children and elderly people [20]. It usually colonizes the pharynx, and more rarely the genital mucosa. Infections caused by this bacterial species are highly contagious. The most common causative agent (53.4%) in maternal sepsis-related death from 2010 through 2016 was *S. pyogenes* according to some Japanese authors [40]. GAS diseases have been reported to increase over time in Canada and the United Kingdom, and new reports focusing on increasing invasive cases also appear. According to the saying “only if you seek you will find”, in 2015, Public Health England registered about 1900 cases of GAS bacteremia, while 50 invasive GAS cases were reported to the Instituto Superiore di Sanità, which hosts a voluntary reporting tool for Italy. The combination of new genes encoding high virulence associated with antibiotic resistance in circulating strains plus a host with reduced defenses to infection can lead to dramatic development in serious GAS diseases [1,40].

The newest developments in whole-genome sequencing technology allow a fairly detailed characterization of *GAS* clinical isolates including genes encoding virulence factors, especially *emm* type and toxin production, as well as others for antibiotic resistance. The use of recent technologies can characterize invasive GAS isolates and will provide invaluable information on population dynamics and strain features associated with emerging lineages, virulence factor distributions, spreading of resistance, and vaccine targets in various geographic areas [8,41].

**Table 2:** Distribution of *S. pyogenes* antibiotic resistance in different countries/regions according to recent data.

	Country	(Study Period) Total n	Antimicrobial Resistance				Ref.
Europe			Macrolide	Lincosamide	Tetracycline	Qionolone	
	Bulgaria	(2013–2016) n = 329	23–40%	-	-	-	[14]
	Greece	(2018–2023) n = 52	20.4%	18.7%	40.8%	2%	[42]
	Spain	(2007–2020) n = 1983	8.7%	3.9%	12.0%	-	[43]
	Hungary	(2008–2017) n = 1104	10.5%	9.2%	-	13.5%	[44]
	Russia	(2014–2017) n = 792	12.1–17.2%	2.4%	-	0.3–0.8%	[45]
North and South America	USA	(2016–2017) n = 3873	16–23%	-	22.6%	1.4%	[41]
	Brazil	(2008–2012) n = 92	14.3%	15.4%	20.9%	0%	[46]
Asia and Australia	China	(2020–2021) n = 114	94.74%	92.98%	87.72%	-	[47]
	China	(2009–2016) n = 140	93.5%	94.2%	86.4%	-	[48]
	Japan	(2007–2008; 2012; 2018) n = 634	34.9–60%	-	-	11.5–14.3%	[49]
	Taiwan	(2000–2019) n = 320	18.1–58.4%	6–58.4%	-	-	[6]
	Australia	(2007–2021) n = 318	6%	-	10%	0%	[50]
Africa	Northwest Ethiopia	(2020) n = 14	21.4%	50%	14.3%	7.2%	[51]
	Southwest Ethiopia	(2013) n = 355	0%	0%	52.5%	-	[52]
Middle East and North Africa region	Cyprus, Saudi Arabia, Egypt, etc.	(1995–2015) review	Ranged from 1.1%–12–70%	-	-	-	[15]

**Table 3:** Distribution of *S. agalactiae* antibiotic resistance in different countries/regions according to recent data.

	Country	(Study Period) Total n	Antimicrobial Resistance				Ref.
Europe			Macrolide	Lincosamide	Tetracycline	Qionolone	
	Bulgaria	(2018–2019) n = 107	58.88%	15.89%	94.62%	10.28%	[17]
	Denmark	(2005–2018) n = 1875	8.1% (2007) 23.8% (2010)	6.5% (2006) 20.4% (2009)	-	-	[4]
	Denmark	(2018–2019) n = 101	21.0%	26.0%	-	-	[53]
	France	(2007–2019) n = 1262	21.0%	-	91.0%	-	[54]
	France	(2007–2014) n = 8757	36.2%	26.3%	86.5%	0.8%	[55]
	Germany	(2009–2010) n = 978	22.4%	14.1%	-	-	[56]
	Iceland	(1976–2015) n = 118	9.0%	1.0%	81.6%	0%	[57]
	Portugal	(2005–2015) n = 218	16.1%	14.2%	85.8%	-	[58]
	Portugal	(2009–2015) n = 555	35.1%	33.9%	-	0.5%	[59]
	Serbia	(2009–2016) n = 432	23.1%	-	86.0%	0%	[12]
	Serbia	(2015–2020) n = 1071	26.7%	22.1%	85.2%	0%	[60]
	Spain	(2010–2016) n = 242	21.5%	17.6%	-	-	[61]
North and South America	USA	(2008–2016) n = 21,250	54.8%	43.2%	83.9%	2.3%	[62]
	Nicaragua	(2019–2020) n = 85	37.6%	31.7%	-	0%	[7]
Asia	Iran	(2017) n = 27	44.4%	29.6%	-	11.1%	[63]
	China	(2008–2015) n = 193	74.1%	64.2%	68.9%	-	[64]
	China	(2015–2017) n = 304	78.3%	68.2%	80.1%	-	[65]
	Taiwan	(2006–2015) n = 225	48.9%	51.4%	-	-	[66]
	Taiwan	(2003–2017) n = 182	68.1%	65.9%	-	-	[67]
Africa	21 countries	(1989–2019) n = 4564	20.82%	19.63%	82.6%	24.56%	[68]

##### *S. agalactiae* (GBS) Infections

*S. agalactiae* (GBS) is the second most common cause of streptococcal infections after GAS. It can cause various severe infections such as meningitis, bacteremia, and sepsis in infants, which can be life-threatening. GBS is also responsible for invasive infections in elderly and immune-compromised adults with various medical conditions such as cirrhosis, diabetes, breast cancer, decubitus ulcer, and neurogenic bladder. This bacterium possesses many virulence factors similar to GAS such as capsules, adhesins, exotoxins, enzymes, and invasins, which can inhibit immune response. GBS commonly causes skin and soft-tissue infections and osteomyelitis, and more rarely endocarditis and pneumonia. In younger patients, it can also cause uro-genital infections either alone or as a co-infectious agent [1,69]. Globally, GBS causes more than 300,000 cases of neonatal disease, including bacteremia (78%), meningitis (16%), and pneumonia (15%), with a fatality rate of over 8%, resulting in 90,000 infant deaths every year. About 19.7 million pregnant women were found to have recto-vaginal GBS colonization in 2020. In the same period, more than 394,000 infants were diagnosed with invasive GBS cases, of which 231,800 were early-onset (infections occurring within the first 7 days of life) and 162,200 were late-onset (occurring between 7 days and 89 days after birth) [70]. The main risk factor for prenatal-onset GBS disease and/or invasive complications in infected mothers is maternal GBS colonization during late pregnancy, especially after the emergence and fast development of resistance to antimicrobials in *S. agalactiae* in combination with more virulent capsular serotypes [17,69]. In recent years, a rapid increase in the incidence of various infections, including invasive infections due to GBS, has been reported. During the period of 1990–2017, a more than double increase in frequency was observed in all age groups, with the highest increase recorded in patients aged 65–79, with a mortality rate of up to 25% [1]. The reasons for this trend have not been elucidated and vaccines are still under development [70,71]. The primary reason is likely the rapidly developing multi-drug resistance (MDR) of GBS to at least three antimicrobial groups in recent years [17,69].

#### 2.1.3. Infections Due to Viridans Streptococci

The members of the Streptococci viridans group are part of the oral microbiota, but they are related to several types of infections described in recent times. Locally, *Streptococcus mutans*, alone or in combination with oral *Lactobacillus* species, is a major reason for the development of dental caries due to dental plaque and cariogenic biofilm formation and strong production of acid products after degradation of sugars in the mouth [72]. The pathogens *S. mutans* and oral lactobacilli are named cariogenic bacteria of oral flora because they are directly associated with the progression of dental caries [72,73]. 

When low virulent viridans streptococci such as *S. mitis*, *S. oralis*, *S. intermedius*, *S. sanguinis*, *S. anginosus*, *S. salivarius*, *S. bovis*, and *S. mutans* enter the blood stream after invasive dental procedures, they can cause infective endocarditis. These bacteria have strong adhesins on their cell wall surfaces that allow them to adhere to cardiac prostheses or damaged endocardium, leading to biofilm formation in patients with certain heart conditions or valve prostheses. This type of bacteremia caused by oral streptococci is challenging to eliminate with antibiotics in high-risk hosts, especially when antimicrobial resistance occurs. It is a biofilm-mediated infection that is difficult to treat and can even lead to death [72,73]. Moreover, some streptococcal species, like *S. mitis*, *S. infantis*, and *S. oralis*, frequently exchange genetic information with *S. pneumoniae*. *S. mitis* acts as an external genetic reservoir for pneumococci and receives many genetic elements from the pneumococcal genome [23].

### 2.2. Evolution of Antibiotic Resistance in Streptococcus genus

#### 2.2.1. Alterations in PBPs and Susceptibility to Penicillin and Other Beta-Lactams

Beta-lactam antibiotics are the first line of choice for the treatment of streptococcal infections, especially in children and pregnant women, as recommended by the Clinical and Laboratory Standards Institute (CLSI) (https://clsi.org /accessed on 2 April 2024) and the European Commission on Antimicrobial Susceptibility Testing (EUCAST) (https://www.eucast.org /accessed on 2 April 2024) guidelines and according WHO criteria (https://www.who.int/news-room/fact-sheets/detail/antibiotic-resistance /accessed on 2 April 2024) [19]. That is why streptococcal susceptibility to penicillin is so crucially important.

**Figure 2:**
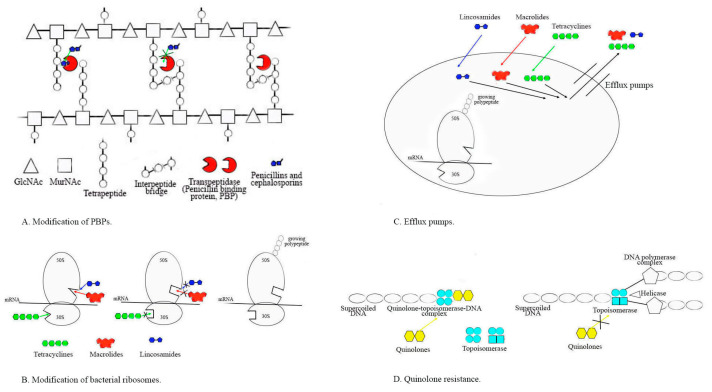
The major mechanisms of resistance in the *Streptococcus* genus. (**A**) Alterations in PBPs (penicillin-binding proteins); (**B**) Bacterial ribosomal target modification by rRNA methylases or due to mutations in the rRNA chain; (**C**) The presence of active efflux pumps; (**D**) Changes in DNA gyrase (Topoisomerase) after mutations in the *parC* and/or *gyrA* gene.

Resistance to beta-lactams in pneumococci and viridans streptococci is mediated by mutations in genes encoding penicillin-binding proteins (*pbp*) and alterations in the PBP binding site (Figure 2A), a place where penicillins and cephalosporins bind [32]. The most common amino acid mutations include N605T in *pbp2×*, which is present in 57.2% of tested pneumococcal isolates and the less frequent mutations I371T in *pbp2×* (in 53.8%) and N609D in *pbp1a* (in 34.6%). The first sporadic *S. pneumoniae* and other streptococcal isolates with intermediate resistance were reported in Australia, New Guinea, South Africa, and the United States in the mid-1960s to 1970s [8]. Among 90.5% of penicillin-resistant *S. pneumoniae* (PRSP) isolates carried mutations in PBPs, indicating that mutations were significantly associated with this resistance. These mutations lower the affinity for penicillin to inhibit the final steps of peptidoglycan synthesis by binding to the PBPs. PRSP can survive and multiply even during and after antibiotic treatment, and can also share antibiotic-resistant determinants with each other via transformation [3,32]. PRSP has spread worldwide and has become resistant to other antimicrobials such as macrolides, tetracyclines, and chloramphenicol, making it multi-drug-resistant (MDR) [30]. In the period 2000–2015, PRSPs were found in a low range of 3.8 to 14.3% in the USA and Turkey [29,31]. In Poland, 45% of PRSPs were reported [21], and in Japan, it ranged from 33.8% and 67% [25,34]. During the same period, penicillin non-susceptibility in causative agents of invasive pneumococcal disease (IPD) was found to be around 20% in Spain and Croatia [28,30], 42.8% in Brazil [33], and 32% in China [35]. Since 2015, this resistance has increased in all pneumococcal isolates up to 39.7% in Argentina [32], 38 to 46.5% in Bulgaria [16,26,74], and more than 80% in Tunisia [36]. 

Antibiotic prophylaxis with amoxicillin is recommended against bacteremia, which occurs during invasive dental procedures due to oral viridans group streptococci and can lead to infective endocarditis in patients (mostly children) with certain heart diseases. The discovery of amoxicillin-resistant strains of viridans group streptococci in the mouths of children carriers (ranging from 5.5% to 86.3%) with heart diseases raises concerns that prophylaxis with amoxicillin may be ineffective [23,72,75,76]. 

The presence of oral viridans streptococci with different levels of resistance to amino-penicillins in samples collected from oral cavities in healthy children or children with co-morbidities, especially *S. mitis*, *S. oralis*, *S. sanguinis*, and *S. salivarius*, was detected. *S. mitis* and *S. sanguinis* showed the most frequently found resistance phenotype, with MIC values of ampicillin and amoxicillin reaching up to 128 µg/mL. Highly resistant *S. oralis* was found more frequently in a study that verified the endodontic content [75,76]. 

*S. pyogenes* is regarded as highly susceptible to the beta-lactam family of antibiotics, 80 years after the introduction of penicillin, with stable MICs of GAS, which has an unclear reason, but since the 1940s, an increasing number of treatment failures have been reported. In the past 20 years, the rate of penicillin failure dramatically increased to almost 40% and became significant after 2000 in some regions of the world [77]. As far as is currently known, the main reasons for penicillin failure are: (i) intracellular persistence of this pathogen due to the poor penetration of beta-lactams into tonsillar tissues; (ii) inactivation of penicillins without an inhibitor from beta-lactamase and protection of GAS due to bacteria producing extracellular beta-lactamase (namely *Staphylococcus aureus*, *Moraxella catarrhalis*, *Haemophilus* spp., and some of the anaerobes that are commonly part of the resident nasopharyngeal microbiota or play a role such as co-infection agent); (iii) frequent instances of co-aggregation between *M. catarrhalis* colonizing the nasopharynx and some M serotype GAS, which may enhance streptococcal adhesion to human epithelial cells; the resulting common biofilm formation could be an important factor in explaining therapeutic failures and recurrences due to the susceptibility to antibiotics of *S. pyogenes* clinical isolates [51,77]. There have been reports of certain strains of GAS in China, Japan, Mexico, India, and Ethiopia that have shown increased MICs to penicillin and cephalosporins near or around breakpoints approaching or exceeding breakpoints [8,9,51]. The criteria for susceptibility to penicillin, and other beta-lactams according to CLSI are MICs ≤ 0.12 mg/mL (https://clsi.org /accessed on 2 April 2024) and according to EUCAST are MICs ≤ 0.25 mg/mL (https://www.eucast.org /accessed on 2 April 2024). Out of 7025 studied strains, the newest mechanisms of beta-lactam resistance in *S. pyogenes* with mutations in the peptidoglycan synthetic enzyme *pbp2x* gene, similar to those in pneumococci and viridans group streptococci (transformations in PBPs), were described in 137 probes (samples). Many of the tested strains demonstrated the possibility of changing in vitro susceptibility to various penicillins and cephalosporins, enabling them to escape antibiotic pressure. In 2022, Beres et al. [8] analyzed 26465 GAS genome sequences and identified amino acid changes in PBP1a, 1b, 2a, and 2x. Although these mutations were found only in a small number of strains, they were associated with multiple *emm* streptococcal types, indicating that they could spread to new hosts and cause invasive infections with high mortality. The new data had shown that mutations such as PBP2x chimeras may lead to reduced susceptibility to penicillins and cephalosporins and increased virulence of GAS [8,9]. The first interspecies horizontal transfer from *S. dysgalactiae* subsp. *equisimilis* donors to *S. pyogenes* of PBP2B and PBP2X resulting in *GAS* strains with a naturally acquired chimeric PBP2X protein have significantly decreased susceptibility to some beta-lactams including penicillin and cephalosporins under positive antibacterial therapy selection [8]. Detailed information on the latest genetic studies on *pbp2x* genes in a very high number of GAS strains and the correlation with MICs to beta-lactams were presented in 2023 by Yu D.et al. [78]. They stated that mutations in GAS PBPs occurred rarely, with less than three amino acid changes. Only four out of 9667 strains contained mutations near the active sites of PBP2x or PBP1a transpeptidase. The authors proposed that GAS with reduced susceptibility to beta-lactams associated with mutations in the pbp2x gene were widespread, but they are still very rare. The reduced susceptibility to penicillin and other beta-lactams in GAS has been demonstrated due to amino acid substitutions within PBPs that affect the ability to bind these antimicrobials (Figure 2A). The highest MICs >2.0 mg/mL of penicillin were reported from Japanese patients with pharyngitis during 2006–2008 (≤0.12 mg/mL, according to CLSI) and from blood and wound samples of GAS isolates in the USA between 2017–2018 (according to CLSI) that showed Ampicillin eight-fold higher and cefotaxime three-fold higher MICs [78]. They concluded that for most GAS infections, beta-lactam antibiotics must be used as the first line of choice for treatment, but ongoing surveillance of the GAS population is in the public health interest and helps clinicians understand the changing nature of medically important bacteria [78].

GBS is still recognized as being universally susceptible to beta-lactam antibiotics; however, some authors have reported transformations in PBPs due to amino acid substitutions in PBP2X, PBP1A, and PBP2b [79]. The prevalence of GBS strains with MICs around susceptibility breakpoints approaching or exceeding breakpoints is high in Japan, which was reported to be 2.3% before 2006 and increased to 14.7% from 2012 through 2013 [80]. Nearly 69% of GBS with increased MICs to penicillin become multi-drug-resistant (MDR). This raises concerns about the use of ampicillin since 1996 in the United States for preventing the vertical transmission of GBS, which could contribute to the development of MDR GBS, especially in Japan [80]. African authors found that at least 55% of GBS isolates from Cameroonian women became MDR [81]. The data presented in the tables are difficult to compare due to some differences in the interpretation guidelines and evolving changes in the various years of the two usage systems, and some authors did not strictly indicate which guidelines they used. Some of the MIC results are significantly higher than the susceptibility breakpoint, but others are around the cutoff values at this time, and this may lead to a bias for lower susceptibility to penicillin results. While vancomycin remains largely effective, there have been a few vancomycin-resistant cases in GBS reported only by Par et al. in 2014, which have not been confirmed from other studies because, in this paper, the concept of “higher MIC” was eventually mixed with “resistance” to vancomycin [82]. The presence of GBS with mutations in *pbp* genes and possibly developing lower susceptibility later to beta-lactams are serious health problems, especially for children and pregnant women [79]. Cefotaxime is recommended as a first-line drug for the treatment of early- and late-onset neonatal sepsis, lung infections, and meningitis in modern neonatology. The development and spread of possible GBS resistance to third-generation cephalosporins, and even more so to vancomycin, are becoming important global problems [63]. The drug options for the prevention and treatment of infections due to GBS are limited, and they are increasingly limited for infections due to MDR GBS [82]. 

#### 2.2.2. Resistance to MLSB Antibiotics

There is an increasing resistance to alternative antibiotics of penicillins such as macrolides and lincosamides among the *Streptococcus genus*, especially in Asia [6,34,35,47]. Macrolide resistance (MR) is the most common type of resistance among pneumococci and beta-streptococci groups A and B. Macrolides are commonly used to treat respiratory tract infections in both children and adults and were used empirically during the COVID-19 pandemic [83,84]. The first MR *S. pneumoniae* isolates were detected in 1967 in Canada [27]. MR rates vary widely depending on geographic regions. The earlier data (before 2010) for European IPD agents on MR varied from 20–26.6% in Spain [30] up to 44.9% in Serbia [37]. Later reports from numerous European studies showed dramatically higher MR for invasive and non-invasive pneumococcal isolates: 47.7% in Turkey [29], 43.9–58.5% in Bulgaria [16,26], 52.9% in Poland [21], 37.5–45.2% in the USA [31], 59.6% in Ethiopia [46], and more than 94% found in China [35] and in Japan [25,34]. The highest level of resistance (92.3%) to clindamycin was detected in recent years in China [35]; in Tunisia it was 64.0% [36], in Turkey 52.4% [29], in Poland 51.0% [21], and in Bulgaria 46.9% [16]. Combined resistance to ß-lactams and macrolides–lincosamides in *S. pneumoniae*, particularly in childhood, has rapidly spread and become a difficult global problem to overcome [3,13].

The first *S. pyogenes* clinical isolate presenting MR was found in the USA in 1968. Authors from Brazil reported that MLSB resistance during 2008–2012 was 14.3–15.4% [42]. From 2013 to date, the incidence of MR dramatically increased up to 20–40%, and lincosamide up to 19% in several European countries, such as Bulgaria and Greece [14,44]. However, GAS isolates have varied widely, both geographically and temporally. In other European countries during 2008–2020, the rate of MR remained low in Hungary and Spain, in the range of 3.9–13.8% [43,84,85].

In some African countries such as Ethiopia, resistant GAS appeared after 2013. At 2020 MR is up to 21% and clindamycin resistance becomes to 50%. In the Middle East and North Africa the results vary widely from 4.2% to 23% in Lebanon and 0.2% to 33.9% in Iran, but from 14.3% in Northwest Ethiopia to 52.5% in Southwest Ethiopia and 70% in Yemen [15,48,51,52]. In the USA, MR has ranged from 16% through 23% [41]. The highest level of resistance is reported in the Asian area. The emergence of MR GAS in Taiwan has been associated with the *emm*12-ST36/*erm*(B) lineage spreading, and has increased from 18% in 2009 to 58% a few years later [6]. In China, MR in combination with *emm*12-ST36/*erm*(B) and *emm*1-ST28/*erm*(B) rose from 15% in 2000 to 95% during the COVID-19 pandemic [27,49]. In Japan, it has been found that distributions of *emm* types *12* and *28* in GAS isolates during different periods are associated with the *erm(B)* gene and the presence of high MR prevalence in 60.9% [86]. A relationship between GAS virulence and MR has emerged in the last few years. The MLS_B_ phenotype and especially *erm*(B) genes are associated with the prevalence of several *emm* types in different periods and regions (*emm*12, 4, 28, 77, 75, 11, 22, 92, 58, 60, 94, 63, 114). Four *emm* clusters (A-C4, E1, E6, E2) have been linked to MR, as well as the increasing cell invasiveness of GAS [27,50,87]. Only in Australia and Russia was the GAS resistance at low levels—MR 6–17.2% and 0–2.4% for clindamycin, respectively [45,62].

The evolution of GBS resistance to macrolides/lincosamides shows an increasing trend during the last decade in the USA, up to 54.8%/43.2% [53], and in most European countries such as Bulgaria (58.88%/15.89%) [17], Denmark (23.8%/26%) [4,58], Portugal (16.1–35.1%/14.2–33.9%) [59,60], and Serbia (23.1–26.7%/22.1%) [12,54]. In contrast, a slight downward trend has been found in France (36.2–21%/26.3%) [55,56], and slower increasing results were reported in Germany (22.4%/14.1%) [61] and Spain (21.5%/17.6%) [65]. Only Iceland has reported a resistance rate of less than 20% to the MLSB group with no increase before the COVID-19 pandemic [65]. This problem is of particular concern, with the invasive GBS isolates reported to be resistant in China and Taiwan with rates ranging from 78% and 49–68% to macrolides and 68% or 51.4–66% to lincosamides before 2017 [64,66,67,68]. African authors from 21 countries reported MLSB resistance of about 20% and tetracycline resistance of more than 82% before 2019 [88]. The use of clindamycin, which is the preferred therapeutic agent for patients with streptococcal pneumonia, empyema, soft tissue abscess, and toxin-mediated infection due to its inhibition of bacterial toxin production, is hindered when resistance is present, and this can be fatal for the patient. An association between increasing antibiotic resistance and the prevalence of type III and V GBS isolates, particularly CC-12 and CC-17 4 strains, was reported in some regions [6,17].

Major mechanisms of MLSB resistance that appear in the *Streptococcus* genus are: (i) ribosomal target enzyme modifications (Figure 2B) caused by rRNA methylases that lead to cross-resistance between macrolides, lincosamides, and streptogramins B (expressed constitutive—cMLSb or inducible—iMLSb phenotype encoded by *erm*-class genes; (ii) ribosomal target alterations (Figure 2B)—mutations in the domain V of 23S rRNA chain or ribosomal L4 and L22 proteins responsible for macrolide resistance in pneumococci and some streptococci; (iii) the presence of active efflux pumps (Figure 2C) causes bacterial resistance to macrolides alone (usually associated with M phenotype), and *mefA*, *mefE*, *msrD* genes defined resistance to 14- and 15-member lactone-ring macrolides, but not to 16-membered macrolides, lincosamides, or to streptogramin B antibiotics; the adenylation of clindamycin due to a nucleotidyl-transferase led to bacterial resistance to clindamycin (*lnu* family genes) [89,90]. MLSB resistance in streptococci is commonly mediated by two classes of methylases determined by chromosomally located genes *erm(B)* (at first identified in *S. sanguinis)* and *erm(TR)*, which is a subclass of *erm(A*), with 82.5% nucleotide identity between them. In contrast to *erm(B)*, which is primarily associated with a cMLSB phenotype and rarely with an iMLSB, genes *erm(TR)* and *erm(A*) were encoding iMLSB phenotype and occasionally some strains with these genes and cMLSB have been reported. While all types of iMLSB isolates are susceptible to lincosamides, those with cMLB are highly resistant to them. Notably, resistance to ketolides is observed in cMLSB and iMLSB-A *S. pyogenes* isolates. The MR gene *erm(B)* blocks the binding of macrolides to ribosomes (antibiotics targeting protein synthesis), while *mef(A)* and *mef(E)* genes produce an efflux pump that regulates the entry of the antibiotics [89,90].

#### 2.2.3. Resistance to Tetracyclines 

Tetracyclines are not commonly used to treat pneumococcal and other streptococcal infections because they are not suitable for use in childhood and in pregnant women to eliminate group B streptococcus. However, there is a high resistance to tetracycline (TR) among Gram-positive and Gram-negative bacteria because this antibiotic is relatively cheap, and is extensively used for prophylaxis and treatment of animals, as well as for the therapy of some human infections around the world [67,68]. Many authors reported increasing TR after 2010 for pneumococcal isolates in various countries. Resistance rates were found to be 39.5% in Bulgaria [16], 43.1% in Poland, [21], 38.6% in Ethiopia [46], 39.5% in Tunisia [36], and 83.7% in China [35]. Moderate levels of resistance were detected at 20.9–22.4% in Brazil and the USA [34,42]. For GAS isolates, problematic TR was found in the range of 40.8% in Greece [44] to 87.72% in China [35,66]. High levels of GBS TR were detected in many countries after 2010 in a range of 68.9–80.1% in China [35] to 82.6% in Africa [69]; Europe: 81.6% in Iceland [65] up to 86% in Serbia [12,54], 85.8% in Portugal [54]; 86.5–91% in France [55,56] and 94.62% (2018–2019) in Bulgaria [17]. The mechanisms of TR in streptococci mainly include ribosomal protection, enzymatic deactivation (Figure 2B), and efflux pumps (Figure 2C), all of which inhibit protein synthesis and are acquired through the acquisition of *tet* genes, with the *tetM* gene being highly prevalent and *tetK*, *tetL*, and *tetO* being less frequent [54,59,65,66,85,91]. Combined TR and MR are usually linked with the insertion of *erm(B)* into the Tn916 transposon carrying *tetM*. This raises serious concerns about the important role of streptococcal and pneumococcal TR strains in the spread of MR strains. The reason for this is that the main source of *tetM* is the easy-to-transfer Tn916 family [23,92]. Integrative conjugative elements in GAS strains are self-replicating DNA segments that can be transferred from cell to cell through direct conjugation, and this mechanism is often used for the horizontal transfer of antibiotic resistance elements such as: *erm(A)*, *erm(B)*, *erm(T)*, *erm(TR)*, *mef(A)*, *msr(D)*, *tet(M)*, and *tet(O)* [93].

#### 2.2.4. Resistance to Fluoroquinolones

The new fluoroquinolones (levofloxacin, moxifloxacin, and gatifloxacin) exhibit strong in vitro activity against members of the *Streptococcus* genus and are successfully used for the treatment of respiratory tract infections in adults and uro-genital tract infections. A new problem has arisen with the emergence of the first Gram-positive cocci with loss of sensitivity to the respiratory quinolones. The level of this resistance in pneumococci has been low in recent years [26,28,33,36]. The first reports for fluoroquinolone-non-susceptible *GAS strains* in Belgium presented a significant increase from 4.3% (2008) to 21.6% (2010) due to reserpine-sensitive efflux and mutations in topoisomerase genes *parC* and *gyrA* [94]. Clonality was determined by *emm* typing with a significant increase in *emm*6 strains among fluoroquinolone-non-susceptible *GAS* [94]. Increasing resistance to this group (antimicrobial class) has been reported in Asia. Before 2020 it was between 11.5–14.3% for Japanese GAS isolates [86] and 11% for Iranian GBS isolates [47]. However, reports from China indicate that there has been a dramatic increase in quinolone-resistant GBS isolates in pregnant women, with rates increasing up to 72.9% in 2021 [95]. In Africa, the evolution of this resistance varied between 7.2% for Ethiopian GAS [51] to 24.56% for GBS in this region [69]. Additionally, some European countries reported 10.28% resistant Bulgarian GBS isolates at first, and 13.5% resistant Hungarian GAS strains [17,43]

The mechanism of quinolone resistance (Figure 2D) is based on changes in topoisomerase IV and DNA gyrase which are hetero-tetramer proteins composed of two subunits: DNA gyrase, encoded by genes *gyrA* and *gyrB*, and topoisomerase IV, encoded by the genes *parC* and *parE.* The resistance most commonly develops after a stepwise mutation in determining regions of either the *parC/E* or the *gyrA/B* gene. The combined mutations in the *parC* or *gyrA* gene play the most effective role in the development of high-level resistance to quinolones, as mutations in either the *parC* or the *gyrA* gene alone can lead to low-level quinolone resistance [96].

## 3. Methods

### 3.1. Search Strategy and Inclusion and Exclusion Criteria

The Preferred Reporting Items for Systematic Reviews and Meta-Analyses (PRISMA) are used for structuring and transparent approaches to identifying, screening, and selecting studies for inclusion in a systematic review or meta-analysis [97]. Published English-language articles from the last 10 years were included, with the most recent trends in resistance in various countries worldwide, and single studies from older years. For this purpose, we used four databases: PubMed, Scopus, Web of Science, and Google Scholar with the suitable keywords in the title or abstract searches using all combinations of the following terms: “*Streptococcus* species“, “*Streptococcus pneumoniae*”, “*Streptococcus pyogenes*”, “*Streptococcus agalactiae*”, “*Streptococcus viridans group*“, “streptococcal infections”, “antibacterial resistance”, “streptococcal antibiotic resistance”, and “mechanisms of resistance”.

### 3.2. Quality Assessment and Data Extraction

After the initial screening, the selected articles were analyzed based on the inclusion and exclusion criteria. To assess the quality of the selected studies, both reviewers independently evaluated the methodologies and results presented in each article. 

### 3.3. Characteristics of Eligible Studies

The selected studies covered a diverse range of streptococcal infections and problematic types of antibiotic resistance in the genus *Streptococcus*. Data about the resistance of streptococcal isolates from 36 geographic regions (Figure 1) were included and analyzed in the present review. This review provides a comprehensive analysis of the dynamics of streptococcal antimicrobial resistance, which become problematic for the eradication of pathogens causing treatment failure and specific resistance mechanisms identified in streptococci to date.

The review presents up-to-date information on in vitro susceptibilities to antibiotics suitable for the treatment of streptococcal infections, as well as the geographical distribution and levels of streptococcal antimicrobial resistance predominantly over the last 15–20 years. Genetic elements encoding different resistance mechanisms and their association with pathogen serotypes and/or infection types are indicated and discussed.

## 4. Conclusions

The results for antibiotic resistance are quite alarming, and urgently call for monitoring of this problem of immense magnitude and implementation of precautionary measures for controlling the spread of resistant mutant strains. For most streptococcal infections, especially in children and pregnant women, beta-lactam antibiotics must remain the first-line choice for the treatment, but the use of non-personalized empiric broad-spectrum antimicrobial therapy promotes the spread of MDR streptococcal etiologic agents. This fact creates a vicious circle that must be overcome by antibiotic policy. Studying the evolution of this process and the mechanisms responsible for the spread may help to find ways to slow the progression of resistance and discover alternative promising therapies for serious diseases due to the genus *Streptococcus*.

## Data Availability

Data are contained within the review.

## References

[1] Creti R. (2017). Have group A and B streptococcal infections become neglected diseases in Europe?. Eur. J. Clin. Microbiol. Infect. Dis..

[2] Carapetis J.R., Steer A.C., Mulholland E.K., Weber M. (2005). The global burden of group A streptococcal diseases. Lancet Infect. Dis..

[3] Fan S., Duan N., Chen W., Zhao X., Wang L., Du P., Guo J. (2023). Genomic Epidemiology of *Streptococcus pneumoniae* Isolated in a Tertiary Hospital in Beijing, China, from 2018 to 2022. Pathogens.

[4] Slotved H.C., Hoffmann S. (2020). The Epidemiology of Invasive Group B Streptococcus in Denmark from 2005 to 2018. Front. Public Health.

[5] Stevens D.L., Bryant A.E., Ferretti J.J., Stevens D.L., Fischetti V.A. (2022). Streptococcus pyogenes Impetigo, Erysipelas, and Cellulitis. Streptococcus pyogenes: Basic Biology to Clinical Manifestations [Internet].

[6] Tsai W.C., Shen C.F., Lin Y.L., Shen F.C., Tsai P.J., Wang S.Y., Lin Y.S., Wu J.J., Chi C.Y., Liu C.C. (2021). Emergence of macrolide-resistant *Streptococcus pyogenes emm12* in southern Taiwan from 2000 to 2019. J. Microbiol. Immunol. Infect..

[7] Alemán T., Vielot N.A., Herrera R., Velasquez R., Berrios T., Toval-Ruíz C., Téllez E., Herrera A., Aguilar S., Becker-Dreps S. (2022). Rectovaginal Colonization with Serotypes of Group B *Streptococci* with Reduced Penicillin Susceptibility among Pregnant Women in León, Nicaragua. Pathogens.

[8] Beres S.B., Zhu L., Pruitt L., Olsen R.J., Faili A., Kayal S., Musser J.M. (2022). Integrative Reverse Genetic Analysis Identifies Polymorphisms Contributing to Decreased Antimicrobial Agent Susceptibility in *Streptococcus pyogenes*. mBio.

[9] Yu D., Zheng Y., Yang Y. (2020). Is There Emergence of β-Lactam Antibiotic-Resistant *Streptococcus pyogenes* in China?. Infect. Drug Resist..

[10] Jin Z., Li J., Zhou H., Wang Z., Yi L., Liu N., Du J., Chang C.Y., Ji W. (2022). Serotype Distribution, Virulence Determinants and Antimicrobial Susceptibility of *Streptococcus agalactiae* Isolated from Young Infants. Pathogens.

[11] Gajic I., Mijac V., Opavski N., Stanojevic M., Lazarevic I., Åmitran A., Hadnadjev M., Ranin L. (2014). Distribution of macrolide-resistant genes among isolates of macrolide-resistant *Streptoccocus pyogenes* and *Streptococcus pneumonia*e in Serbia. Arch. Biol. Sci..

[12] Gajic I., Plainvert C., Kekic D., Dmytruk N., Mijac V., Tazi A., Glaser P., Ranin L., Poyart C., Opavski N. (2019). Molecular epidemiology of invasive and non-invasive group B Streptococcus circulating in Serbia. Int. J. Med. Microbiol..

[13] de Miguel S., Pérez-Abeledo M., Ramos B., García L., Arce A., Martínez-Arce R., Yuste J., Sanz J.C. (2023). Distribution of Multidrug-Resistant Invasive Serotypes of *Streptococcus pneumoniae* during the Period 2007–2021 in Madrid, Spain. Antibiotics.

[14] Muhtarova A., Gergova R., Mitov I. (2017). Distribution of macrolide resistance mechanisms in Bulgarian clinical isolates of *Streptococcus pyogenes* during the years of 2013-2016. J. Glob. Antimicrob. Resist..

[15] Rafei R., Hawli M., Osman M., Dabboussi F., Hamze M. (2020). Distribution of *emm* types and macrolide resistance determinants among group A streptococci in the Middle East and North Africa region. J. Glob. Antimicrob. Resist..

[16] Alexandrova A., Pencheva D., Setchanova L., Gergova R. (2022). Association of pili with widespread multidrug-resistant genetic lineages of non-invasive pediatric *Streptococcus pneumoniae* isolates. Acta Microbiol. Immunol. Hung..

[17] Gergova R., Muhtarova A., Tsitou V.M., Mitov I. (2021). Emergence of multidrug-resistant and-hypervirulent *Streptococcus agalactiae* in Bulgarian patients. Balkan Med. J..

[18] Lin J.N., Chang L.L., Lai C.H., Huang Y.H., Chen W.F., Yang C.H., Hsu J., Lin H.H., Chen Y.H. (2015). High prevalence of fluoroquinolone-nonsusceptible *Streptococcus pyogenes emm12* in Taiwan. Diagn. Microbiol. Infect. Dis..

[19] World Health Organization (2021). A Report about Antibiotic Resistance. https://www.who.int/news-room/fact-sheets/detail/antibiotic-resistance.

[20] Gergova R., Petrova G., Gergov S., Minchev P., Mitov I., Strateva T. (2016). Microbiological features of the upper respiratory tract infections in Bulgarian children for the period 1998-2014 our university’s experience. Balk. Med. J..

[21] Niedzielski A., Korona-Glowniak I., Malm A. (2013). High prevalence of *Streptococcus pneumoniae* in adenoids and nasopharynx in preschool children with recurrent upper respiratory tract infections in Poland--distribution of serotypes and drug resistance patterns. Med. Sci. Monit..

[22] Brealey J.C., Chappell K.J., Galbraith S., Fantino E., Gaydon J., Tozer S., Young P.R., Holt P.G., Sly P.D. (2017). Streptococcus pneumoniae colonization of the nasopharynx is associated with increased severity during respiratory syncytial virus infection in young children. Respirology.

[23] Andam C.P., Hanage W.P. (2015). Mechanisms of genome evolution of Streptococcus. Infect. Genet. Evol..

[24] Dagan R., Danino D., Weinberger D.M. (2022). The Pneumococcus-Respiratory Virus Connection—Unexpected Lessons from the COVID-19 Pandemic. JAMA Netw. Open.

[25] Okada T., Sato Y., Toyonaga Y., Hanaki H., Sunakawa K. (2016). Nationwide survey of *Streptococcus pneumoniae* drug resistance in the pediatric field in Japan. Pediatr. Int..

[26] Setchanova L., Murdjeva M., Stancheva I., Alexandrova A., Sredkova M., Stoeva T., Yoneva M., Kurchatova A., Mitov I. (2017). Serotype changes and antimicrobial nonsusceptibility rates of invasive and non-invasive Streptococcus pneumoniae isolates after implementation of 10-valent pneumococcal nontypeable *Haemophilus influenzae* protein D conjugate vaccine (PHiD-CV) in Bulgaria. Braz. J. Infect. Dis..

[27] Berbel D., González-Díaz A., López de Egea G., Càmara J., Ardanuy C. (2022). An Overview of Macrolide Resistance in Streptococci: Prevalence, Mobile Elements and Dynamics. Microorganisms.

[28] Butić I., Gužvinec M., Jelić M., Groš I., Lucić S., Bošnjak M., Tambić A.A. (2022). Serotype distribution and antimicrobial resistance of invasive *Streptococcus pneumoniae* isolates among Croatian adults during a fifteen-year period (2005–2019). Croat. Med. J..

[29] Arvas A., Çokuğraş H., Gür E., Gönüllü N., Taner Z., Tokman H.B. (2017). Pneumococcal nasopharyngeal carriage in young healthy children after pneumococcal conjugate vaccine in Turkey. Balkan Med. J..

[30] Sempere J., González-Camacho F., Domenech M., Llamosí M., Del Río I., López-Ruiz B., Gimeno M., Coronel P., Yuste J. (2022). A national longitudinal study evaluating the activity of cefditoren and other antibiotics against non-susceptible *Streptococcus pneumoniae* strains during the period 2004-20 in Spain. J. Antimicrob. Chemother..

[31] Suaya J., Mendes R., Sings H., Arguedas A., Reinert R., Jodar L., Isturiz R.E., Gessner B.D. (2020). *Streptococcus pneumoniae* serotype distribution and antimicrobial non susceptibility trends among adults with pneumonia in the United States, 2009–2017. J. Infect..

[32] von Specht M., García Gabarrot G., Mollerach M., Bonofiglio L., Gagetti P., Kaufman S., Vigliarolo L., Toresani I., Lopardo H.A. (2021). Resistance to β-lactams in *Streptococcus pneumoniae*. Rev. Argent. Microbiol..

[33] Caierão J., Hawkins P., Sant’anna F.H., da Cunha G.R., d’Azevedo P.A., McGee L., Dias C. (2014). Serotypes and genotypes of invasive *Streptococcus pneumoniae* before and after PCV10 implementation in southern Brazil. PLoS ONE.

[34] Kawaguchiya M., Urushibara N., Aung M.S., Kudo K., Ito M., Sumi A., Kobayashi N. (2021). Clonal lineages and antimicrobial resistance of nonencapsulated *Streptococcus pneumoniae* in the post-pneumococcal conjugate vaccine era in Japan. Int. J. Infect. Dis..

[35] Fu J., Yi R., Jiang Y., Xu S., Qin P., Liang Z., Chen J. (2019). Serotype distribution and antimicrobial resistance of *Streptococcus pneumoniae* causing invasive diseases in China: A meta-analysis. BMC Pediatr..

[36] Ayadi M.B., Mehiri E., Draoui H., Ghariani A., Essalah L., Raoult D., Fournier P.E., Slim-Saidi L.N. (2020). Phenotypic and molecular characterization of macrolide resistance mechanisms among *Streptococcus pneumoniae* isolated in Tunisia. J. Med. Microbiol..

[37] Mijac V., Opavski N., Markovic M., Gajic I., Vasiljevic Z., Sipetic T., Bajcetic M. (2015). Trends in macrolide resistance of respiratory tract pathogens in the paediatric population in Serbia from 2004 to 2009. Epidemiol. Infect..

[38] Gergova R., Muhtarova A., Mitov I., Setchanova L., Mihova K., Kaneva R., Markovska R. (2019). Relation between *emm* types and virulence gene profiles among Bulgarian *Streptococcus pyogenes* clinical isolates. Infect. Dis..

[39] Ikebe T., Tominaga K., Shima T., Okuno R., Kubota H., Ogata K., Chiba K., Katsukawa C., Ohya H., Tada Y. (2015). Increased prevalence of group A streptococcus isolates in streptococcal toxic shock syndrome cases in Japan from 2010 to 2012. Epidemiol. Infect..

[40] Tanaka H., Katsuragi S., Hasegawa J., Tanaka K., Osato K., Nakata M., Murakoshi T., Sekizawa A., Kanayama N., Ishiwata I. (2019). The most common causative bacteria in maternal sepsis-related deaths in Japan were group A Streptococcus: A nationwide survey. J. Infect. Chemother..

[41] Li Y., Rivers J., Mathis S., Li Z., Velusamy S., Nanduri S.A., Van Beneden C.A., Snippes-Vagnone P., McGee L., Chochua s. (2020). Genomic Surveillance of *Streptococcus pyogenes* Strains Causing Invasive Disease, United States, 2016-2017. Front. Microbiol..

[42] Arêas G.P., Schuab R.B., Neves F.P., Barros R.R. (2014). Antimicrobial susceptibility patterns, emm type distribution and genetic diversity of *Streptococcus pyogenes* recovered in Brazil. Mem. Inst. Oswaldo Cruz.

[43] Gajdács M., Ábrók M., Lázár A., Burián K. (2020). Beta-Haemolytic Group A, C and G Streptococcal Infections in Southern Hungary: A 10-Year Population-Based Retrospective Survey (2008-2017) and a Review of the Literature. Infect. Drug. Resist..

[44] Meletis G., Soulopoulos Ketikidis A.L., Floropoulou N., Tychala A., Kagkalou G., Vasilaki O., Mantzana P., Skoura L., Protonotariou E. (2023). Antimicrobial resistance rates of *Streptococcus pyogenes* in a Greek tertiary care hospital: 6-year data and literature review. New Microbiol..

[45] Butler T.A.J., Story C., Green E., Williamson K.M., Newton P., Jenkins F., Varadhan H., van Hal S. (2024). Insights gained from sequencing Australian non-invasive and invasive *Streptococcus pyogenes* isolates. Microb. Genom..

[46] Sharew B., Moges F., Yismaw G., Abebe W., Fentaw S., Vestrheim D., Tessema B. (2021). Antimicrobial resistance profile and multidrug resistance patterns of *Streptococcus pneumoniae* isolates from patients suspected of pneumococcal infections in Ethiopia. Ann. Clin. Microbiol. Antimicrob..

[47] Rostami S., Moeineddini L., Ghandehari F., Khorasani M.R., Shoaei P., Ebrahimi N. (2021). Macrolide-resistance, capsular genotyping and associated factors of group B *Streptococci* colonized pregnant women in Isfahan, Iran. Iran J. Microbiol..

[48] Tesfaw G., Kibru G., Mekonnen D., Abdissa A. (2015). Prevalence of group A β-haemolytic Streptococcus among children with pharyngitis in Jimma town, Southwest Ethiopia. Egypt. Soc. Ear Nose Throat Allied Sci..

[49] Lu B., Fang Y., Fan Y., Chen X., Wang J., Zeng J., Li Y., Zhang Z., Huang L., Li H. (2017). High Prevalence of Macrolide-resistance and Molecular Characterization of *Streptococcus pyogenes* Isolates Circulating in China from 2009 to 2016. Front. Microbiol..

[50] Muhtarova A., Mihova K., Markovska R., Mitov I., Kaneva R., Gergova R. (2019). Molecular *emm* typing of Bulgarian macrolide-resistant *Streptococcus pyogenes* isolates. Acta Microbiol. Immunol. Hung..

[51] Kebede D., Admas A., Mekonnen D. (2021). Prevalence and antibiotics susceptibility profiles of *Streptococcus pyogenes* among pediatric patients with acute pharyngitis at Felege Hiwot Comprehensive Specialized Hospital, Northwest Ethiopia. BMC Microbiol..

[52] Rafei R., Al Iaali R., Osman M., Dabboussi F., Hamze M. (2022). A global snapshot on the prevalent macrolide-resistant *emm* types of Group A Streptococcus worldwide, their phenotypes and their resistance marker genotypes during the last two decades: A systematic review. Infect. Genet. Evol..

[53] Francois Watkins L.K., McGee L., Schrag S.J., Beall B., Jain J.H., Pondo T., Farley M.M., Harrison L.H., Zansky S.M., Baumbach J. (2019). Epidemiology of Invasive Group B *Streptococcal* Infections Among Nonpregnant Adults in the United States, 2008-2016. JAMA Intern. Med..

[54] Kekic D., Gajic I., Opavski N., Kojic M., Vukotic G., Smitran A., Boskovic L., Stojkovic M., Ranin L. (2021). Trends in molecular characteristics and antimicrobial resistance of group B *Streptococci*: A multicenter study in Serbia, 2015–2020. Sci. Rep..

[55] Plainvert C., Hays C., Touak G., Joubrel-Guyot C., Dmytruk N., Frigo A., Poyart C., Tazi A. (2020). Multidrug-Resistant Hypervirulent Group B Streptococcus in Neonatal Invasive Infections, France, 2007–2019. Emerg. Infect. Dis..

[56] Hays C., Louis M., Plainvert C., Dmytruk N., Touak G., Trieu-Cuot P., Poyart C., Tazi A. (2016). Changing Epidemiology of Group B Streptococcus Susceptibility to Fluoroquinolones and Aminoglycosides in France. Antimicrob. Agents Chemother..

[57] López Y., Parra E., Cepas V., Sanfeliú I., Juncosa T., Andreu A., Xercavins M., Pérez J., Sanz S., Vergara A. (2018). Serotype, virulence profile, antimicrobial resistance and macrolide-resistance determinants in *Streptococcus agalactiae* isolates in pregnant women and neonates in Catalonia, Spain. Enferm. Infect. Microbiol. Clin..

[58] Slotved H.-C., Jens K.M., Mohammad R.K., Stine Y.N. (2021). The serotype distribution of *Streptococcus agalactiae* (GBS) carriage isolates among pregnant women having risk factors for early-onset GBS disease: A comparative study with GBS causing invasive infections during the same period in Denmark. BMC Infect. Dis..

[59] Martins E.R., Pedroso-Roussado C., Melo-Cristino J., Ramirez M. (2017). Portuguese Group for the Study of Streptococcal Infections. Streptococcus agalactiae Causing Neonatal Infections in Portugal (2005–2015): Diversification and Emergence of a CC17/PI-2b Multidrug Resistant Sublineage. Front. Microbiol..

[60] Lopes E., Fernandes T., Machado M.P., Carriço J.A., Melo-Cristino J., Ramirez M., Martins E.R. (2018). Portuguese Group for the Study of Streptococcal Infections. Increasing macrolide resistance among *Streptococcus agalactiae* causing invasive disease in non-pregnant adults was driven by a single capsular-transformed lineage, Portugal, 2009 to 2015. Euro. Surveill..

[61] Lohrmann F., Berg A., Wicker E., Imm A., Krause G., Zürn K., Berner R., Hufnagel M., Lander F. (2021). Prevalence of capsular serotype, pilus island distribution, and antibiotic resistance in pediatric and adult invasive group B Streptococcus isolates: Data from a nationwide prospective surveillance study in Germany. Pediatr. Infect. Dis. J..

[62] Ivanchik N.V., Sukhorukova M.V., Chagaryan A.N., Dekhnich A.V., Kozlov R.S., Andreev V.A., Bekker G.G., Varganova A.N., Gudkova L.V., Ershova M.G. (2020). Antimicrobial resistance of clinical *Streptococcus pyogenes* isolates in Russia: The results of multicenter epidemiological study. PEHASus 2014–2017. Antimicrob. Agents Chemother..

[63] Boscarino G., Romano R., Iotti C., Tegoni F., Perrone S., Esposito S. (2024). An Overview of Antibiotic Therapy for Early- and Late-Onset Neonatal Sepsis: Current Strategies and Future Prospects. Antibiotics.

[64] Ji W., Liu H., Madhi S.A., Cunnington M., Zhang Z., Dangor Z., Zhou H., Mu X., Jin Z., Wang A. (2019). Clinical and Molecular Epidemiology of Invasive Group B Streptococcus Disease among Infants, China. Emerg. Infect. Dis..

[65] Björnsdóttir E.S., Martins E.R., Erlendsdóttir H., Haraldsson G., Melo-Cristino J., Ramirez M., Kristinsson K.G. (2019). Group B Streptococcal Neonatal and Early Infancy Infections in Iceland, 1976-2015. Pediatr. Infect. Dis. J..

[66] Lu B., Chen X., Wang J., Wang D., Zeng J., Li Y., Li D., Zhu F., Cui Y., Huang L. (2016). Molecular characteristics and antimicrobial resistance in invasive and noninvasive Group B Streptococcus between 2008 and 2015 in China. Diagn. Microbiol. Infect. Dis..

[67] Tsai M.H., Hsu J.F., Lai M.Y., Lin L.C., Chu S.M., Huang H.R., Chiang M.C., Fu R.H., Lu J.J. (2019). Molecular Characteristics and Antimicrobial Resistance of Group B Streptococcus Strains Causing Invasive Disease in Neonates and Adults. Front. Microbiol..

[68] Kao Y., Tsai M.H., Lai M.Y., Chu S.M., Huang H.R., Chiang M.C., Fu R.H., Lu J.J., Hsu J.F. (2019). Emerging serotype III sequence type 17 group B streptococcus invasive infection in infants: The clinical characteristics and impacts on outcomes. BMC Infect. Dis..

[69] Gizachew M., Tiruneh M., Moges F., Adefris M., Tigabu Z., Tessema B. (2019). *Streptococcus agalactiae* from Ethiopian pregnant women; prevalence, associated factors and antimicrobial resistance: Alarming for prophylaxis. Ann. Clin. Microbiol. Antimicrob..

[70] Donders G.G., Halperin S.A., Devlieger R., Baker S., Forte P., Wittke F., Slobod K.S., Dull P.M. (2016). Maternal Immunization with an Investigational Trivalent Group B Streptococcal Vaccine: A Randomized Controlled Trial. Obstet. Gynecol..

[71] Edwards M.S., Rench M.A., Rinaudo C.D., Fabbrini M., Tuscano G., Buffi G., Bartolini E., Bonacci S., Baker C.J., Margarit I. (2016). Immune Responses to Invasive Group B Streptococcal Disease in Adults. Emerg. Infect. Dis..

[72] Ahirwar S.S., Gupta M.K., Snehi S.K. (2019). Dental caries and lactobacillus: Role and ecology in the oral cavity. Int. J. Pharm. Sci. & Res..

[73] Ribeiro S.M., Bueno P.C.P., Cavalheiro A.J., Klein M.I. (2023). Effect of Extracts, Fractions, and Isolated Molecules of *Casearia sylvestris* to Control *Streptococcus mutans* Cariogenic Biofilm. Antibiotics..

[74] Setchanova L., Alexandrova A., Pencheva D., Sirakov I., Mihova K., Kaneva R., Mitov I. (2018). Rise of multidrug-resistant *Streptococcus pneumoniae* clones expressing non-vaccine serotypes among children following introduction of the 10-valent pneumococcal conjugate vaccine in Bulgaria. J. Glob. Antimicrob. Resist..

[75] Nemoto H., Nakano K., Masuda K., Wada K., Ardin A.C., Nomura R., Ooshima T. (2011). Distribution of oral streptococci highly resistant to amoxicillin in dental plaque specimens from Japanese children and adolescents. J. Med. Microbiol..

[76] Masuda K., Nemoto H., Nakano K., Naka S., Nomura R., Ooshima T. (2012). Amoxicillin-resistant oral streptococci identified in dental plaque specimens from healthy Japanese adults. J. Cardiol..

[77] Cattoir V., Ferretti J.J., Stevens D.L., Fischetti V.A. (2016). Mechanisms of Antibiotic Resistance. Streptococcus pyogenes: Basic Biology to Clinical Manifestations [Internet].

[78] Yu D., Guo D., Zheng Y., Yang Y. (2023). A review of penicillin binding protein and group A *Streptococcus* with reduced-β-lactam susceptibility. Front. Cell. Infect. Microbiol..

[79] Hayes K., O’Halloran F., Cotter L. (2020). A review of antibiotic resistance in Group B *Streptococcus*: The story so far. Crit. Rev. Microbiol..

[80] Seki T., Kimura K., Reid M., Miyazaki A., Banno H., Jin W., Wachino J., Yamada K., Arakawa Y. (2015). High isolation rate of MDR group B *Streptococci* with reduced penicillin susceptibility in Japan. J. Antimicrob. Chemother..

[81] Djuikoue C.I., Djoulako P.D.D., Wouambo R.K., Foutsa R.Y., Ngatcheu D.E., Apalata T. (2022). Frequency and Antibiotic Susceptibility Patterns of *Streptococcus agalactiae* Strains Isolated from Women in Yaounde, Cameroon. Microbiol. Res..

[82] Park C., Nichols M., Schrag S.J. (2014). Two cases of invasive vancomycin-resistant group B streptococcus infection. N. Engl. J. Med..

[83] Li H., Zhou L., Zhao Y., Ma L., Zhang H., Liu Y., Liu X., Hu J. (2023). Epidemiological analysis of Group A streptococcus infection diseases among children in Beijing, China under COVID-19 pandemic. BMC Pediatr..

[84] Ruiz-Garbajosa P., Cantón R. (2021). COVID-19: Impact on prescribing and antimicrobial resistance. Rev. Esp. Quimioter..

[85] Villalón P., Bárcena M., Medina-Pascual M.J., Garrido N., Pino-Rosa S., Carrasco G., Valdezate S. (2023). National Surveillance of Tetracycline, Erythromycin, and Clindamycin Resistance in Invasive *Streptococcus pyogenes*: A Retrospective Study of the Situation in Spain, 2007–2020. Antibiotics.

[86] Ubukata K., Wajima T., Morozumi M., Sakuma M., Tajima T., Matsubara K., Itahashi K., Iwata S. (2020). Changes in epidemiologic characteristics and antimicrobial resistance of *Streptococcus pyogenes* isolated over 10 years from Japanese children with pharyngotonsillitis. J. Med. Microbiol..

[87] Bi S., Xu M., Zhou Y., Xing X., Shen A., Wang B. (2019). A multicomponent vaccine provides immunity against local and systemic infections by Group A Streptococcus across serotypes. mBio.

[88] Gizachew M., Tiruneh M., Moges F., Tessema B. (2019). *Streptococcus agalactiae* maternal colonization, antibiotic resistance and serotype profiles in Africa: A meta-analysis. Ann. Clin. Microbiol. Antimicrob..

[89] Iannelli F., Santoro F., Santagati M., Docquier J.-D., Lazzeri E., Pastore G., Cassone M., Oggioni M.R., Rossolini G.M., Stefani S. (2018). Type M Resistance to Macrolides Is Due to a Two-GeneEfflux Transport System of the ATP-Binding Cassette (ABC) Superfamily. Front. Microbiol..

[90] Liu Z., Jiang X., Li J., Ji W., Zhou H., Gong X., Miao B., Meng S., Duan L., Shi Q. (2023). Molecular characteristics and antibiotic resistance mechanisms of clindamycin-resistant *Streptococcus agalactiae* isolates in China. Front. Microbiol..

[91] Mudzana R., Mavenyengwa R.T., Gudza-Mugabe M. (2021). Analysis of virulence factors and antibiotic resistance genes in group B streptococcus from clinical samples. BMC Infect. Dis..

[92] Ousmane S., Diallo B.A., Ouedraogo R. (2018). Genetic determinants of tetracycline resistance in clinical *Streptococcus pneumoniae* serotype 1 isolates from Niger. Antibiotics.

[93] Jespersen M.G., Lacey J.A., Tong S.Y.C., Davies M.R. (2020). Global genomic epidemiology *of Streptococcus pyogenes*. Infect. Genet. Evol..

[94] Van Heirstraeten L., Leten G., Lammens C., Goossens H., Malhotra-Kumar S. (2012). Increase in fluoroquinolone non-susceptibility among clinical *Streptococcus pyogenes* in Belgium during 2007–10. J. Antimicrob. Chemother..

[95] Wang J., Zhang Y., Lin M., Bao J., Wang G., Dong R., Zou P., Chen Y., Li N., Zhang T. (2023). Maternal colonization with group B Streptococcus and antibiotic resistance in China: Systematic review and meta-analyses. Ann. Clin. Microbiol. Antimicrob..

[96] Kargar M., Moein Jahromi F., Doosti A., Handali S. (2014). Molecular Investigation of Quinolone Resistance of Quinolone Resistance-Determining Region in *Streptococcus pneumoniae* Strains Isolated from Iran Using Polymerase Chain Reaction-Restriction Fragment Length Polymorphism Method. Osong Public Health Res. Perspect..

[97] Page M.J., McKenzie J.E., Bossuyt P.M., Boutron I., Hoffmann T.C., Mulrow C.D., Shamseer L., Tetzlaff J.M., Akl E.A., Brennan S.E. (2021). The PRISMA 2020 statement: An updated guideline for reporting systematic reviews. BMJ.

